# Regulation of the *Escherichia coli* HipBA Toxin-Antitoxin System by Proteolysis

**DOI:** 10.1371/journal.pone.0039185

**Published:** 2012-06-15

**Authors:** Sonja Hansen, Marin Vulić, Jungki Min, Tien-Jui Yen, Maria A. Schumacher, Richard G. Brennan, Kim Lewis

**Affiliations:** 1 Antimicrobial Discovery Center, Department of Biology, Northeastern University, Boston, Massachusetts, United States of America; 2 Department of Biochemistry, Duke University School of Medicine, Durham, North Carolina, United States of America; University of Florida, United States of America

## Abstract

Bacterial populations produce antibiotic-tolerant persister cells. A number of recent studies point to the involvement of toxin/antitoxin (TA) modules in persister formation. *hipBA* is a type II TA module that codes for the HipB antitoxin and the HipA toxin. HipA is an EF-Tu kinase, which causes protein synthesis inhibition and dormancy upon phosphorylation of its substrate. Antitoxins are labile proteins that are degraded by one of the cytosolic ATP-dependent proteases. We followed the rate of HipB degradation in different protease deficient strains and found that HipB was stabilized in a *lon^-^* background. These findings were confirmed in an *in vitro* degradation assay, showing that Lon is the main protease responsible for HipB proteolysis. Moreover, we demonstrated that degradation of HipB is dependent on the presence of an unstructured carboxy-terminal stretch of HipB that encompasses the last 16 amino acid residues. Further, substitution of the conserved carboxy-terminal tryptophan of HipB to alanine or even the complete removal of this 16 residue fragment did not alter the affinity of HipB for *hipBA* operator DNA or for HipA indicating that the major role of this region of HipB is to control HipB degradation and hence HipA-mediated persistence.

## Introduction

Bacterial populations stochastically produce a small number of non-growing persister cells that are tolerant to antibiotics [1]–[4]. Persisters are phenotypic variants that are genetically identical to the susceptible cells within a clonal population. Thus, persistence is a non-heritable, transient trait [5], [6]. Previously, we have shown that the recalcitrance of biofilms is largely due to the presence of persisters [7], [8]. Recent studies by our group link persistence to chronic infectious disease. In the case of cystic fibrosis patients infected with *Pseudomonas aeruginosa*, high persister (*hip*) mutants are selected for over the course of infection [4]. Similarly, *Candida albicans hip* mutants are selected in patients with oral thrush [9]. These findings indicate that persisters are largely responsible for failure to eradicate chronic infections [4].

Non-growing persisters make up a small part of the population: 10^−6^ to 10^−4^ in exponentially growing cultures and ∼10^−2^ in stationary phase [3], [8]. Selection of *Escherichia coli* for mutants with increased persister formation produced a strain with two point mutations in *hipA*, G22S and D291A, (*hipA7* allele) [10]–[14]. *hipA* is the toxin of the *hipBA* toxin/antitoxin (TA) pair. The antitoxin HipB represses the *hipBA* operon by cooperative binding to four operator sites [10], [11], [15] and inactivates the toxin HipA. Ectopic expression of HipA causes multidrug tolerance [16]. Despite the strong phenotype of the gain-of-function allele *hipA7* and HipA overexpression, a deletion of *hipA* did not produce a phenotype [12], [16]–[19]. Similarly, ectopic expression of two other toxins, RelE and MazF, also strongly increased tolerance to antibiotics, whereas a deletion of the toxin gene had no phenotype [20]. This is not surprising given the considerable redundancy in these mRNA interferases – *E. coli* has at least 10 of them. Importantly, progressive deletion of all ten mRNase loci caused a pronounced decrease in the persister fraction [21].

One notable exception to the redundancy phenomenon is the toxin TisB, a membrane-acting peptide that causes dormancy by decreasing the pmf. Deletion of the type I TA module (small RNA antitoxin/protein toxin) *tisAB* led to a pronounced decrease in the level of persisters. TisB is induced by the SOS response, and becomes the main mechanism of persister formation during SOS response, so a deletion has a phenotype [22], [23].

Unlike any other toxins of type II TA modules (protein antitoxin/protein toxin) which so far group mainly into either gyrase inhibitors (ParD, CcdB), mRNA interferases (RelE, MazF, YoeB, HicA, Doc) [24]–[26] or PIN domain fold proteins (VapC) [22], [27], HipA is a kinase with a eukaryotic Ser/Thr kinase-like fold [16]. Replacing the conserved amino acids in the phosphorylation site (S150A) or the Mg^2+^- or catalytic binding sites (D332Q and D309Q respectively) abolishes the ability to confer growth arrest and antibiotic tolerance [16]. Elongation factor Tu (EF-Tu) was identified as a HipA target which points to a likely mechanism of HipA-mediated persister formation [15]. HipA phosphorylates EF-Tu, and Thr^382^-phosphorylated EF-Tu leads to stasis since it can no longer bind aminoacyl-tRNA [15]. Under the standard regime of batch culture growth the persistence function of HipA is masked by its tight interaction with HipB. To activate HipA, the antitoxin HipB has to be removed or degraded. Proteolytic regulation of the antitoxin has been demonstrated for several TA modules. In *E. coli*, the chromosomally encoded TA modules MazEF, RelBE, YefM/YoeB, HicAB, DinJ/YafQ and MqsRA are regulated by the AAA+ ATP-dependent proteases Lon and/or ClpPX [24], [28]–[30]. ClpPA degrades the PhD antitoxin of the plasmid-encoded PhD/Doc TA module [22]. The other two ATP-dependent proteases HslVU and FtsH do not have any known antitoxin substrates [22], [24]. In this study we provide evidence that Lon is the main protease responsible for HipB degradation. Our data suggests that Lon recognizes the unstructured C-terminus of HipB.

## Results

### Rapid degradation of HipB is dependent on the presence of Lon

**Figure 1:**
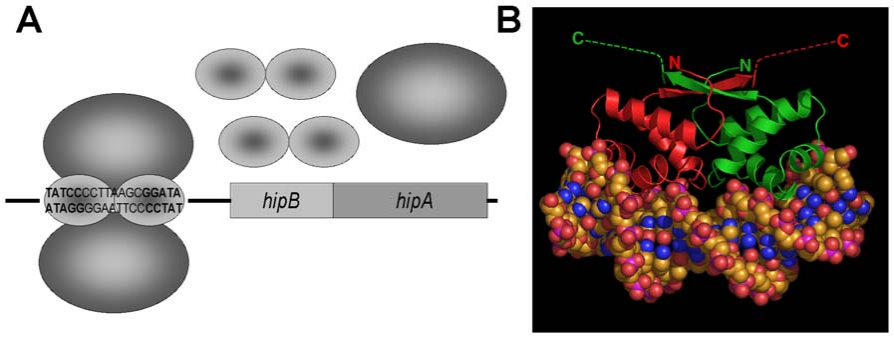
Overview of the *hipBA* locus of *E.* coli based on Schumacher *et al*. (A) Model of the *hipBA* operon. One of four operator sites is shown. (B) View of the crystal structure of the HipB dimer bound to a 21 base pair *hipBA* operator site (from reference [15]. One HipB subunit is colored green and the other red. The α helices are shown as coils and the α strands as arrows. The amino termini of each subunit are labelled N and the carboxy termini, C. The 16 C-terminal residues (73–88) are unstructured and residues 75–88, which are disordered in the structure of the HipA-HipB-DNA complex, are depicted as dashes and could easily extend from the body of HipB by more than 50 Å.

**Figure 2:**
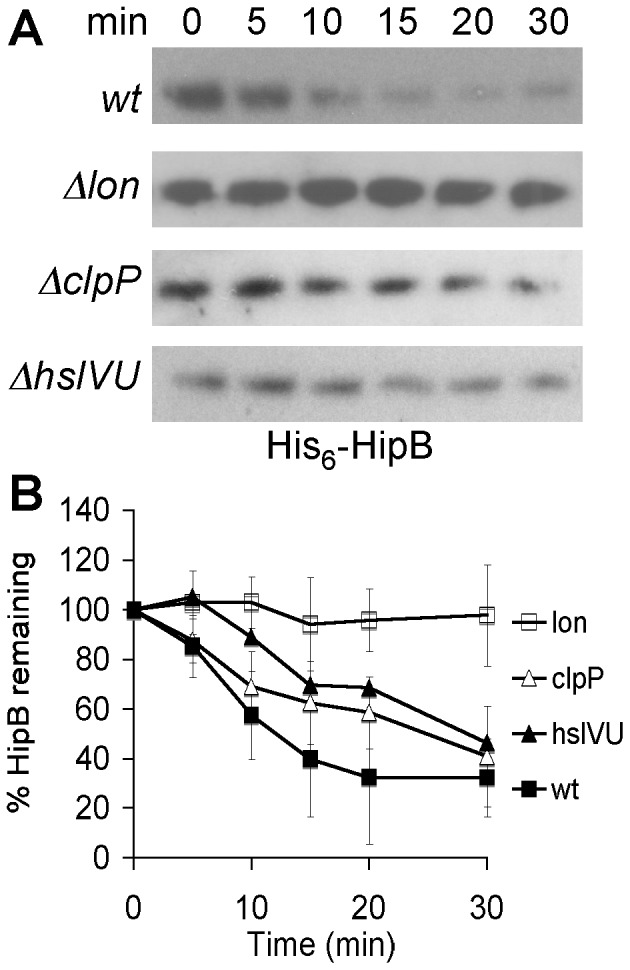
HipB proteolysis in *E. coli* wild type and protease deficient strains. HipB was expressed from pBR*hipB* in BW25113 (KLE901) and its *lon::kan* (KLE902), *clpP::kan* (KLE903) or *hslVU::FRT* (KLE904) derivate. The strains were grown in LB medium, and at an OD_600_ of 0.3 1 mM IPTG was added. After 1 h induction, protein synthesis was inhibited by the addition of 100 µg/ml Cam, and samples for Western blots were removed over the course of 30 min. (A) The presence of HipB in whole cell lysates was detected with an anti-his antibody. (B) The rate of degradation was calculated from at least 3 independent experiments. Closed squares, KLE901 (wild type); open squares, KLE902 (Δ*lon*); closed triangles KLE904 (Δ*hslVU*); open triangles, KLE903 (Δ*clpP*).

HipB does not share homology with any of the known antitoxins. Neutralization of its cognate toxin also differs mechanistically from other TA modules. Typically, the antitoxin contains an extended C-terminal stretch, which is structured only when in complex with its toxin [31]–[36]. Contacts usually involve residues near the active site of the toxin, which can simply be blocked by the antitoxin [33], [35]–[37] or make it sterically impossible for the toxin to reach its target [31], [32], [34]. HipB, however, does not make any contacts with HipA near the active site. One HipB dimer binds two HipA molecules involving interactions with both the N and the C domain of HipA [15] (Fig. 1A). The C terminus of HipB is disordered (Fig. 1B) and remains unstructured in the presence of HipA [15]. To test whether proteolytic regulation is a shared characteristic of HipB with the typical antitoxins of the mRNA interferase and gyrase inhibitor TA modules, despite functional and structural differences, we measured the rate of *in vivo* degradation of HipB in wild type *E. coli*. Since endogenous HipB could not be detected by Western Blotting using a polyclonal antibody to HipB (data not shown), N-terminally six-his tagged HipB (His_6_-HipB) was expressed from a plasmid containing an IPTG inducible promoter (pBR*hipB*). After 60 min of induction, protein synthesis was stopped by the addition of chloramphenicol and the rate of HipB proteolysis was determined by Western blotting (Fig. 2A). His_6_-HipB was degraded with a t_1/2_ of ≈17 min in wild type cells confirming a rate of degradation characteristic for antitoxins [38], [39]. Next, we transformed pBR*hipB* into protease deficient strains lacking *lon* (KLE902); *clpP* (KLE903); or *hslVU* (KLE904) to identify a protease responsible for HipB degradation. We compared the rate of *in vivo* degradation of HipB in wild type to the rate of degradation in the protease deficient strains. Deletion of *clpP* or *hslVU* had a slight effect on HipB. The half life time of HipB was approximately 24 min in Δ*clpP* and 28 min in Δ*hslVU*. Deletion of *lon* stabilized HipB (Fig. 2) (t_1/2_>200 min), indicating that Lon is likely the main protease involved in HipB degradation *in vivo*. Since deletion of Lon protease had the strongest effect on the HipB turnover, we focused our studies on Lon dependent HipB degradation.

### HipB is a substrate of the ATP-dependent protease Lon

**Figure 3:**
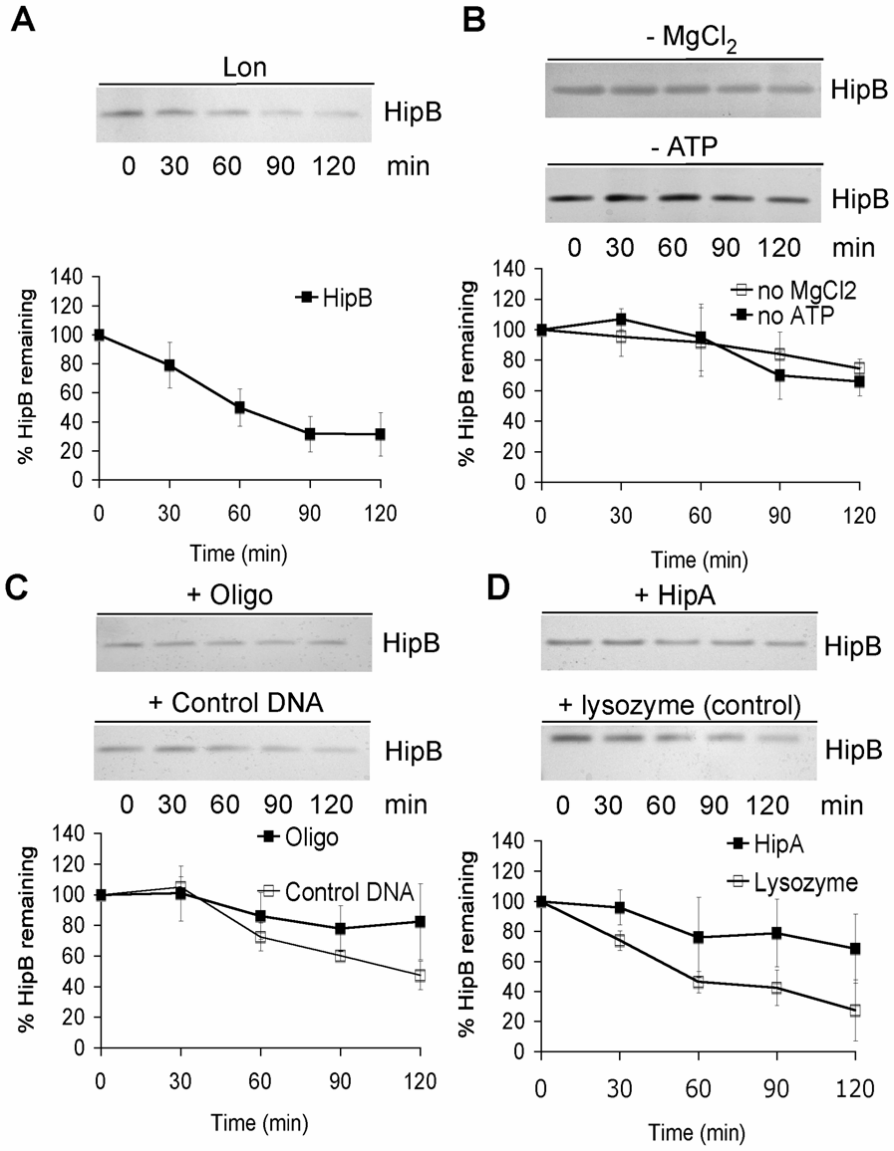
Lon degradation of HipB *in vitro*. 0.6 µM His_6_-Lon and 0.48 µM His_6_-HipB were incubated in reaction buffer at 37°C (50 mM Tris-HCl (pH 8.0), 4 mM ATP, 7.5 mM MgCl_2_) for indicated times with or without the component specified and subjected to SDS-PAGE and silver staining followed by analysis (at least 3 independent experiments were used to calculate HipB turn over). (A) *In vitro* degradation of His_6_-HipB by His_6_-Lon. (B) ATP or MgCl_2_ were omitted in the assay. Closed squares, no ATP; open squares no MgCl_2_. (C) Addition of an oligodeoxynucleotide encompassing the 21 bp *hip* operator (closed squares) or control oligo (open squares) and (D) addition of His_6_-HipA (closed squares) or control protein (lysoszyme) (open squares) to the degradation assay.

To determine whether HipB is directly recognized and degraded by Lon we purified His_6_-Lon and His_6_-HipB for *in vitro* degradation studies. Lon degraded HipB with a t_1/2_ of ≈74 min *in vitro* confirming our findings obtained *in vivo* (Fig. 3A). The HipB decay however was much slower in the *in vitro* assay than *in vivo*. A difference between *in vivo* and *in vitro* degradation rate was also described for the antitoxin RelB which was degraded rapidly (t_½_≈15 min) in a Lon dependent manner *in vivo*, whereas the *in vitro* half life time was >60 min [38], [39]. Substrate degradation by Lon requires ATP hydrolysis and Mg^2+^ [40]. HipB degradation was indeed dependent on the presence of ATP and MgCl_2_ in the reaction buffer demonstrating that degradation of HipB is specific to the addition of active enzyme to the buffer (Fig. 3B). The half-life of HipB in the *in vitro* assay without either MgCl_2_ or ATP was 250 min and 168 min, respectively. There was some residual degradation in the absence of either ATP or Mg2+; however, both factors were clearly required for Lon-dependent degradation of HipB.

HipB functions as an inhibitor of HipA and as an autoregulator of the *hipBA* operon by cooperatively binding to the consensus sequence TATCCN_8_GGATA. We tested whether Lon also degrades HipB when it is bound to DNA or HipA. Addition of a 21 bp oligo ACTATCCCCTTAAGGGGATAG (sequence of top strand) spanning the *hipBA* operator sequence reduced the rate of degradation (t_1/2_>200 min) (Fig. 3C), while addition of a control oligo (sequence of top strand ATGATGAGCTTTCAGAAGATC) showed little effect. Similarly, addition of purified His_6_-HipA slowed down the rate of HipB proteolysis (t_1/2_>200 min) as compared to a control protein (lysozyme) (Fig. 3D). Thus, HipB appears to be rapidly degraded only when it is free and not functioning as a transcriptional inhibitor of the *hipBA* operon or neutralizing the HipA protein.

### The unstructured C-terminus of HipB is critical for degradation

**Figure 4:**
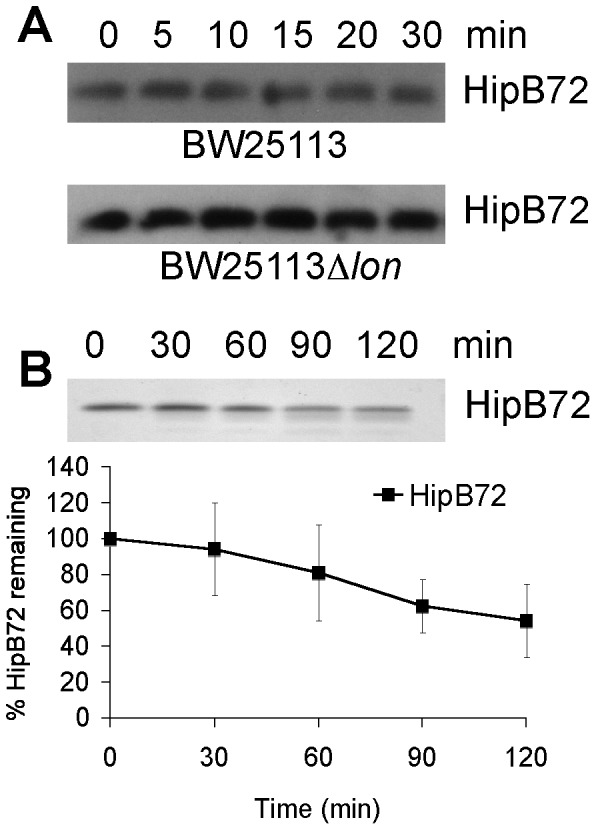
The 16 C-terminal amino acid residues of HipB are required for degradation. (A) Degradation of HipB72 *in vivo*. HipB72 was expressed from a pBR*lac_i_tac* promoter in BW25113 (KLE905) and its *lon::kan* derivate (KLE906). Both strains were grown in LB medium, and at an OD_600_ of 0.3 1 mM IPTG was added. After 1 h of induction, protein synthesis was inhibited by the addition of 100 µg/ml Cam, and samples for Western blots were removed over the course of 30 min. (B) Degradation of HipB 72 *in vitro*. His_6_-HipB72 was purified and added to the Lon degradation assay. At least 3 independent experiments were performed to calculate HipB72 turnover.

**Figure 5:**
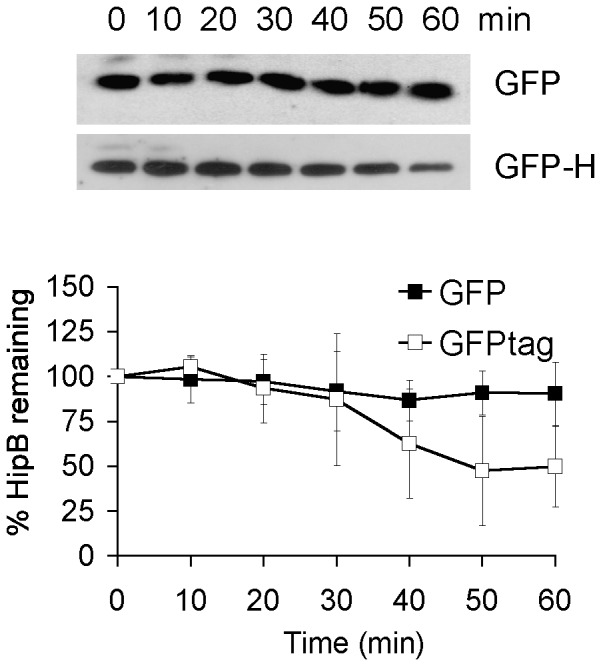
*In vivo* degradation of GFP and a GFP-HipB hybrid. GFP and GFP with C-terminal fusion to the C terminus of HipB were expressed from a pBR*lac_i_tac* promoter in BW25113 (KLE907 and KLE908, respectively). The strains were grown in LB medium, and at an OD_600_ of 0.3 1 mM IPTG was added. After 1 h of induction, protein synthesis was inhibited by the addition of 100 µg/ml Cam, and samples for Western blots were removed over the course of 60 min. Closed squares, GFP; open squares GFP-H (GFP-HipB(73–88)). The graph represents the average of five independent experiments.

The HipB dimerization interface is composed of a small hydrophobic core and the β-lid, a two-stranded intermolecular β sheet that is followed by an unstructured 16 amino acid C-terminus (AKNASPESTEQQNLEW) [15]. Proteases typically bind disordered regions of their substrate, thus the unstructured C terminus appears to be an excellent recognition site for protease attack [15]. To test the hypothesis that the 16 residue C-terminal stretch is critical for degradation, we cloned a truncated HipB (HipB72) lacking the last 16 residues of HipB into pBR creating pBR*hipB72*. We measured the rate of *in vivo* degradation of HipB72 in wild type and Δ*lon* (KLE905 and KLE906, respectively) (Fig. 4). Interestingly, HipB72 is indeed substantially more stabile (t_1/2_>200 min) than full length HipB in wild type indicating that the unstructured C terminus of HipB is essential for degradation by Lon protease (Fig. 4A). As expected, full length HipB72 is also stable in Δ*lon* background. We purified the truncated HipB (His_6_-HipB72) and tested it in the Lon *in vitro* degradation assay. The effect was also noticeable though less pronounced *in vitro*. The half-life time of HipB changed from 74 min for full length HipB to 130 min in the mutant (Fig. 4B). To confirm that the unstructured C terminus of HipB is a degradation signal for Lon protease, we fused the C terminus of GFP with the unstructured C-terminal tail of HipB (creating pBRGFP-H, KLE908), and tested whether addition of the carboxy-terminal stretch of HipB (residues 73–88) causes degradation of GFP, which by itself is stable over the time period of the experiment (t_1/2_>200 min) (Fig. 5). The GFP-HipB tail hybrid was much less stable with a half-life time of ≈53 min confirming that the C-terminus of HipB is critical for rapid proteolysis of HipB.

### The C-terminus of HipB does not affect binding to either HipA or operator DNA

**Figure 6:**
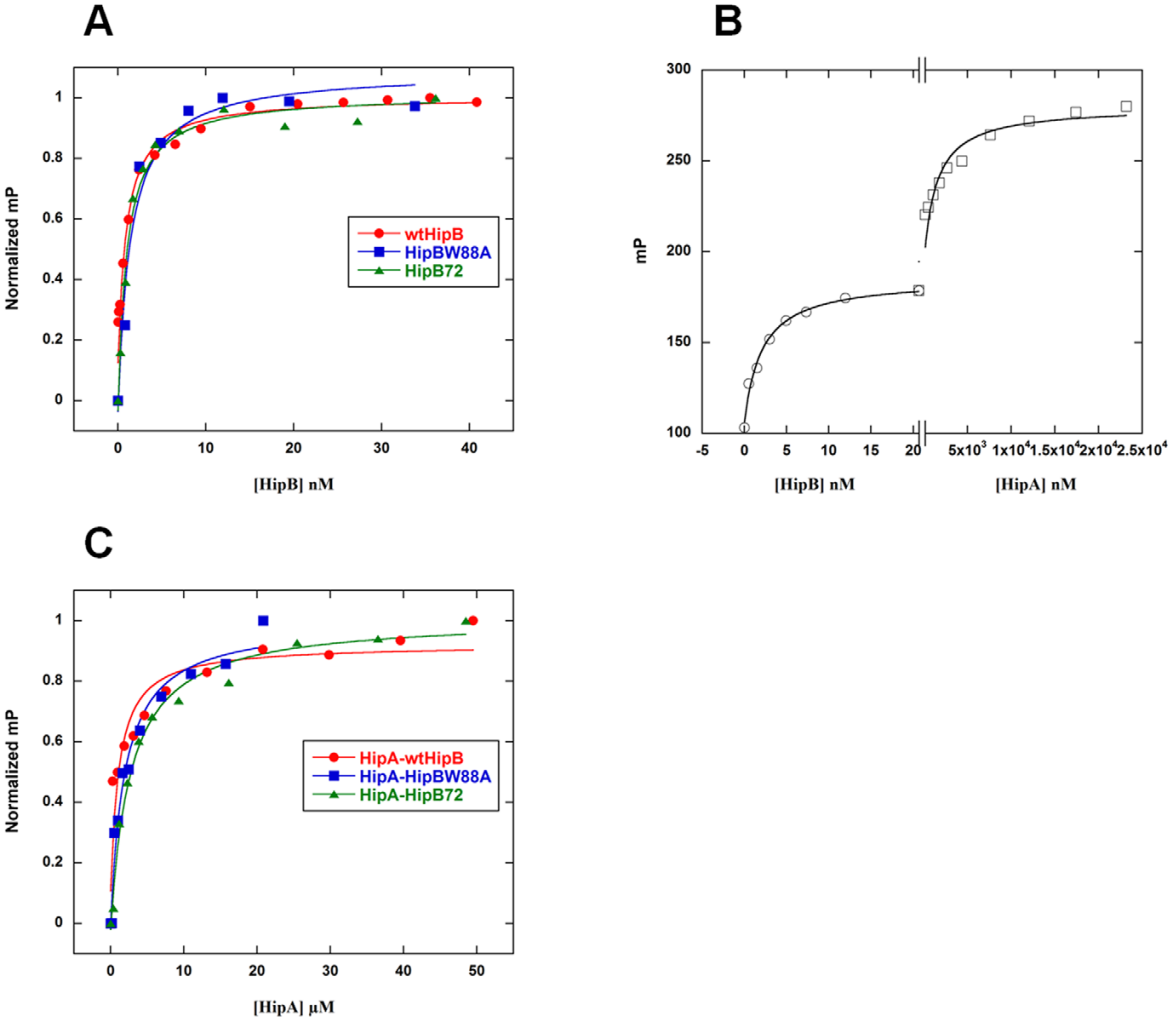
Multiple sequence alignment of selected HipB proteins from a variety of Gram-negative bacteria. CLUSTALW and CLC Main Workbench were used for the alignment and graphic representation, respectively. HipB sequences were downloaded from NCBI database.

**Figure 7:**
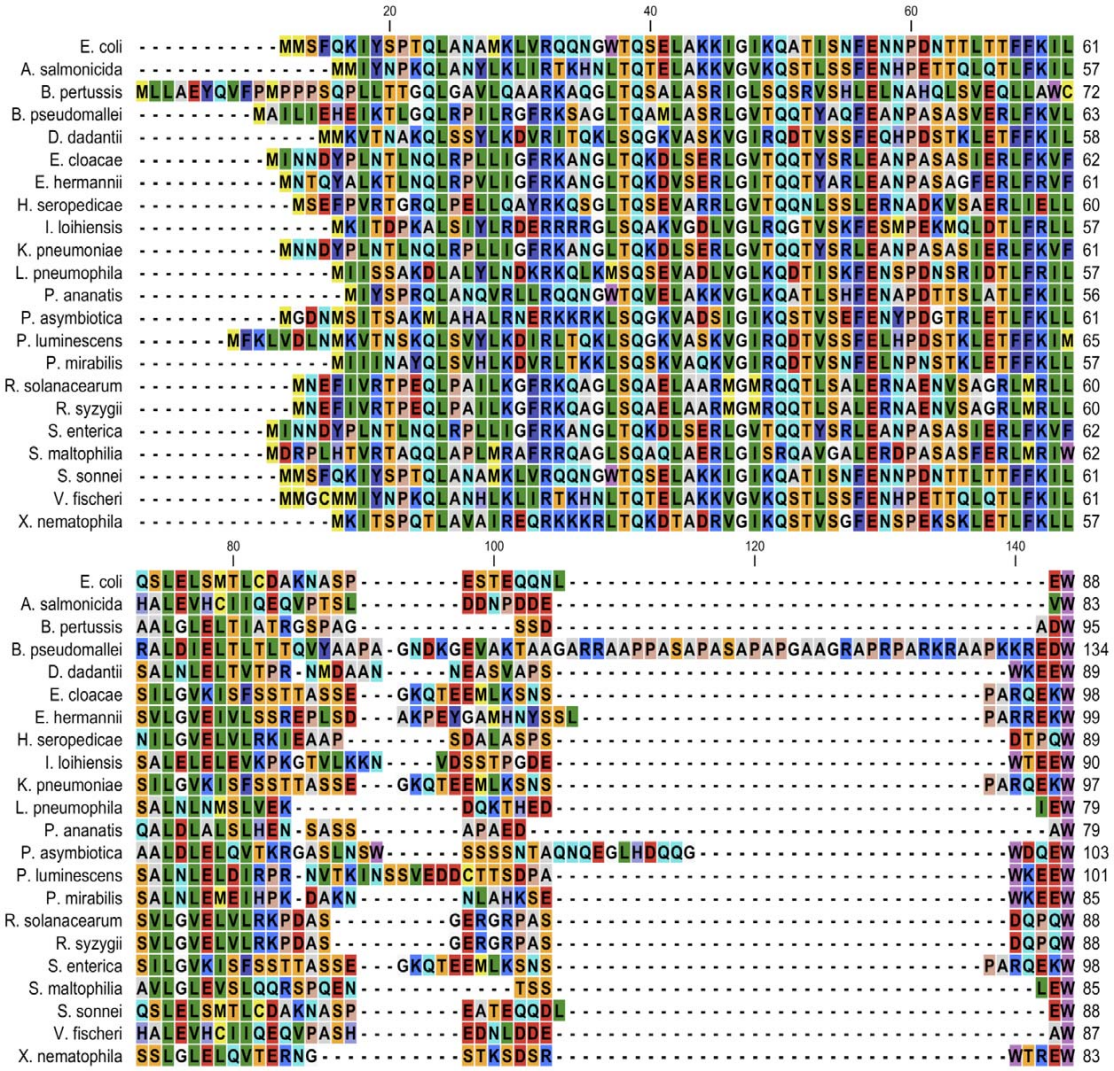
HipB, HipB(W88A) and HipB72 bind *hipBA* operator DNA or HipA identically. (A) Wild type HipB protein (red closed circle), HipB(W88A) protein (blue closed square) or the HipB72 C-terminal truncation protein (green closed triangle) was titrated into fluoresceinated *hipBA O_1_O_2_* operator sequence and the change in fluorescence polarization (normalized millipolarization, mP) plotted as a function of the concentration of the titrant. The typical change in mP of each titration was between 60 and 80 units. The correlation coefficients for each curve fitting were 0.98, 0.99 and 0.99, respectively. (B) Wild type HipA protein was titrated into *hipBA O1O2* DNA after the DNA was prebound by 20 nM wild type HipB monomer. Note that the concentration range is different in the left half and right half of the binding isotherms, with HipB titrations in the nanomolar range and HipA titrations in the micromolar range. (C) Wild type HipA protein was titrated into solutions containing 1 nM fluorescently labelled *hipBA O_1_O_2_* DNA and titrated up to 50 nM wild type HipB monomer, 50 nM HipB(W88A) protein or HipB72 protein. This ensures stoichiometric binding of these HipB proteins to the DNA. Thus, the resulting binding affinity is formally between HipA and HipB that is bound specifically to *hipBA* DNA. The change in mP of each titration was between 88 and 150 units. The correlation coefficients for each curve fitting were 0.95, 0.99 and 0.99, respectively. A representative binding isotherm is shown for each protein binding to DNA or to the HipB-*hipBA O_1_O_2_* complex.

The possibility that the last sixteen residues of HipB had additional functions beyond their role in protein stability was considered especially in light of the finding that the C-terminal residue of HipB, W88, which is universally conserved (Fig. 6), interacted with a small surface pocket of HipA [41]. We first tested the effect of changing residue 88 to an alanine on HipB-DNA affinity. Using a fluorescence polarization-based assay and the *hipBA O_1_O_2_* operator site, we determined that wild type HipB bound this sequence with a K_d_=0.6±0.1 nM (Fig. 7A, 1). As anticipated from the HipA-HipB-DNA crystal structure [15], the HipB(W88A) protein bound this DNA with wild type HipB affinity (K_d_=0.9±0.4 nM). Deletion of the last sixteen residues of HipB, (HipB72) also showed no change in DNA binding affinity (K_d_=0.4±0.1 nM) (Fig. 7A, Table 1).

**Table 1:** Dissociation constants of HipB-DNA and HipA-(HipB-DNA)*.

	*O_1_O_2_* DNA (nM)	HipA (µM)
HipB WT	0.6±0.1	1.0±0.6
HipB(W88A)	0.9±0.4	1.2±0.3
HipB72	0.4±0.1	±0.1

* The binding constants are the average of at least three separate measurements.

To test the hypothesis that W88 or other residues of the C-terminus contributes to HipA binding, we measured the dissociation constant of HipA for HipB (Fig. 7B and C). The reporter molecule in this assay is the *hipBA O_1_O_2_* sequence, which is saturated with HipB by using HipB concentrations 50 fold greater than K_d_. HipA binding to HipB will result in a further, saturable increase in fluorescence polarization from which the HipA-HipB K_d_ can be ascertained. The dissociation constant of HipA binding to HipB was 1 µM under our experimental condition (Fig. 7B and C, Table 1). The K_d_ of HipA for HipB(W88A) was identical to the wild type HipB K_d_ as was the K_d_ of HipA for HipB72 (Fig. 7C, Table 1). Titration of HipA into *O_1_O_2_* DNA in the absence of HipB results in linear, nonspecific DNA binding (data not shown). These results demonstrate that the C-terminus of HipB does not play a role in binding to either *hipBA O_1_O_2_* DNA or HipA.

## Discussion

The *hipBA* toxin/antitoxin locus shares several characteristics with other TA modules, such as the genetic organisation in an operon with the antitoxin overlapping the toxin by one base pair, tight regulation of the operon by the antitoxin and inhibition of the toxin by its antidote. In addition, ectopic expression of the toxin confers growth arrest, which can be overcome by antitoxin expression. However, the HipBA TA system does not group into the three common toxin families of RelBE-, the MazEF- and VapBC-like members. Toxin and antitoxin are structurally and mechanistically distinct from all other characterized TA pairs. HipA is a kinase, and HipB belongs to the Xre-helix-turn-helix family of transcriptional regulators. Binding of HipA-HipB_2_-HipA to DNA introduces a 70° bend in the operator [15]. In contrast to other antitoxins, HipB interacts with HipA via the N and C domain and the C terminus of HipB remains unstructured in the presence of the toxin [15]. Despite functional differences, regulation by proteolysis is a shared characteristic with all other protein-coding antitoxins. We find that HipB is a substrate of Lon protease since HipB is stabilized in the absence of Lon and degraded by Lon *in vitro*. Under standard growth conditions HipB neutralizes HipA and represses transcription of the *hipBA* operon. However, when no new HipB is produced or Lon activity reaches elevated levels, HipB turnover results in free HipA. Shutdown of HipBA synthesis might be further regulated at the transcriptional or translational level, both diminishing the level of HipB decay and thus freeing up HipA. Currently, little is known about the activity of the *hipBA* promoter under different growth conditions. The position of an IHF binding site upstream of the *hip* operon suggests a level of transcriptional regulation beyond repression by HipB and HipBA binding to the operator. The activity of Lon protease is upregulated during stresses [42], [43]. Polyphosphate (poly-P) binds to Lon and promotes degradation of ribosomal proteins (S2, L9, L13) while degradation of other proteins (e.g. SulA) is inhibited by poly-P [44], [45]. Though a regulator of Lon activity that directs Lon to act on HipB has not been identified, it is possible that such a regulator exists. Increased activity of Lon will result in faster degradation of HipB faster and release free HipA. An additional possibility for regulating HipBA is that chaperones can potentially play a role in removing HipB from HipA. Though there is no direct evidence that a HipB is specifically regulated by chaperones, the persister level is 10-fold reduced in a *dnaK* deletion [17]. If a chaperone sequesters HipB, the persister level will be expected to be high due to free HipA. Subsequently, deletion of the chaperone will produce a low persister phenotype.

Irrespective of how it is released, free HipA will in turn phosphorylate EF-Tu and potentially act on additional targets leading to the shutdown of essential cellular functions and thus to dormancy. Phosphorylation of EF-Tu by HipA has been demonstrated only *in vitro* and remains to be confirmed *in vivo*. Additional targets of HipA are likely, and are currently a subject of investigation.

In a recent study, Rotem *et al.*, showed that if the amount of ectopically expressed HipA surpasses a certain threshold growth is arrested, whereas at low HipA levels growth is not affected, which leads to the formation of a distinct dormant; and growing subpopulation, correspondigly [46]. Considering our results, by controlling HipB degradation, Lon protease is the driving factor shifting HipA above or beyond the threshold levels.

Proteolytic degradation of antitoxins generally plays an important role in persister cell formation. Overexpression of Lon protease caused a 70-fold increase in the level of persister cells compared to the wild type [21]. The increase in persistence dropped to a 4-fold difference in a strain lacking all ten mRNA endonuclease TA systems (Δ10) in comparison to wild type control, indicating that Lon-mediated degradation of the antitoxins is responsible for the increase in the persister level [21]. Lon also regulates HipB degradation, and therefore HipBA likely contributes to the increase in persister formation under conditions when Lon is produced and HipB is degraded.

Another Lon substrate is the replication inhibitor CspD [47]. Interestingly, overexpression of CspD causes growth arrest [47] and therefore CspD might also be implicated in persistence. However, its role appears to be minor. The persister fraction in a strain overexpressing CspD increased less than 10-fold, and deletion of *cspD* caused a 2-fold change [48]. Given the known variability observed in the level of persisters, a robust persister phenotype of CspD remains to be established. While HipB degradation by Lon leads to HipA-mediated growth arrest, the situation is reversed for CspD. CspD-mediated growth arrest reversed by Lon, suggesting a possible resuscitation mechanism. It remains to be established how Lon itself is regulated to control toxin degradation.

Our data show that a disordered C-terminus of HipB serves as a degradation signal for the Lon protease. We must note that these results were obtained with an N-terminal his-tagged HipB. It is unlikely however that this N-terminal his-tag affected degradation. The common cause of artifacts in determining degradation of ectopically expressed (including tagged) proteins stems from formation of aggregates, which include the overexpressed protein, chaperones and proteases. This is the case for a number of membrane-associated proteins, whereas overexpression of soluble GST-GFP did not result in aggregation [49]. His_6_-HipB is soluble and we have no indication that aggregates are formed and are interfering with degradation of HipB. HipB and CspD both have in common an extended or disordered C-terminus [47]. While Langklotz and Narberhaus do not observe any differences in stability of CspD point mutations of non-polar residues in the C-terminal region (V73D-A74D) and truncations of the C terminus (lacking the last five, seven, and eleven residues, respectively), we find that a HipB mutant lacking the conformationally flexible C terminus is stabilized suggesting that the C-terminus is a signal sequence for Lon pointing to a different mechanisms of substrate recognition for these two proteins. The possibility that the HipB C-terminus functions beyond acting as a degradation tag has been raised by Evdokimov and colleagues [41]. In the structures of the HipB-HipA and HipA-HipB-DNA complexes [15], HipB residue W88 was found bound in a pocket on the HipA surface approximately 14 Å from the catalytic pocket. This led Evdokimov to suggest that this residue might be important for HipA-HipB interaction as well as potentially acting as an allosteric regulator of HipA activity [41]. Our binding assays show that residue W88 and indeed residues 73 through 88 play no role in HipA binding to HipB as the wild type HipB, HipB(W88A) and HipB72 have identical binding affinities for HipA. Such similar binding affinities would also seem to eliminate the possibility that HipB residue W88 regulates HipA allosterically. However, HipA binding to HipB does appear to block access of Lon to HipB (Fig. 3D). This is not surprising as the (HipA)_2_-HipB dimer structure shows that the C-terminus of HipB is partially shielded from the solvent, which would hinder its access by Lon [15], [41]. Thus, we can conclude that the last sixteen residues of HipB serve as a highly efficient tag for the destruction of the antitoxin HipB by Lon protease but only after its dissociation from the HipA toxin and the *hipBA* operator. This requirement for two dissociation events before HipB degradation might serve to ensure that the switch to persistence is not thrown accidently.

## Materials and Methods

### Bacterial strains and growth conditions

The bacterial strains and plasmids used in this study and their relevant characteristics are listed in Table 2. Strains were grown in LB medium unless otherwise noted. When required, LB broth or LB agar were supplemented with ampicillin (100 µg/ml, Amp) and chloramphenicol (30 µg/ml, Cam).

**Table 2:** Strains and plasmids used in this study.

Strain or plasmid	Relevant characteristics	Source or reference
*E. coli* K-12 strains		
MG1655	F^−^λ^−^ *ilvG rfb-50 rph-1*	[53]
BW25113	*lacI^q^ rrnB3 lacZ4787 hsdR514* Δ*(araBAD)567* Δ*(rhaBAD)568 rph-1*	[54]
BW25113 Δ*lon*	Δ*lon::kan*	Keio collection, [55]
BW25113 Δ*clpP*	Δ*clpP::kan*	Keio collection, [55]
BW25113 Δ*hslVU*	Δ*hslVU::FRT*	This study
KLE901	BW25113 pBRhipB	This study
KLE902	BW25113 Δ*lon* pBRhipB	This study
KLE903	BW25113 Δ*clpP::kan* pBRhipB	This study
KLE904	BW25113 Δ*hslVU::FRT* pBRhipB	This study
KLE905	BW25113 pBRhipB72	This study
KLE906	BW25113 Δ*lon* pBRhipB72	This study
KLE907	BW25113 pBRGFP	This study
KLE908	BW25113 pBRGFP-H	This study
KLE319	MG1655 Δ*hipA* pBADhipA	[16]
AG1 pCA24N*lon*	AG1 *(endA1 recA1 gyrA96 thi-1 relA1 glnV44 hsdR17(r_K_^−^ m_K_^+^)* pCA24N*lon*, GFP free	ASKA library, [56]
KLE909	BL21(DE3)pLysS pEThipA	[41]
KLE910	BL21(DE3)pLysS pEThipB	[15]
KLE911	BL21(DE3)pLysS pEThipB(W88A)	This study
KLE912	BL21(DE3)pLysS pEThipB72	This study
Plasmids		
pBR*lac_i_tac*	bla lacI^q^ Ptac pBR322 ori	[57]
pBAD33	cat araC P_BAD_ pACYC184 ori	[58]
pUA66	pSC101 ori, GFP reporter plasmid carrying GFPmut2, Km^R^	[59]
pCA24N*lon*	pCA24N*lon*, GFP free, Cm^R^	ASKA library, [56]
pBRhipB	pBR*lac_i_tac*-*hipB*	This study
pBRhipB72	pBR*lac_i_tac*-*hipB72*	This study
pBRGFP	pBR*lac_i_tac*-GFP	This study
pBRGFP-H	pBR*lac_i_tac*-GFP*hipB(73–88)*	This study
pBADhipA	pBAD33-*hipA*	[16]
pEThipA	pET28a-*hipA*	[41]
pEThipB	pET15b-*hipB*	[15]
pEThipB(W88A)	pET15b-*hipB w88a*	This study
pEThipB72	pET15b-*hipB72*	This study

### Plasmid constructions

Primers P1hishipBXbaI (CGGTCTAGATAAGGAGATATATGG AATAATGCACCACCACCACCACCACATGAGCTTTCAGAAGATCTA) and P2hipBEcoRI (CCGGAATTCTTACCACTCCAGATTTTGCTG) or P2hipBsEcoRI (CCGG AATTCGTCGCATAGCGTCATTGAGAG) were used to amplify *hipB* from *E. coli* MG1655; P1GFPXbaI (CGGTCTAGATAAGGAGATATATGGAATAATGAGTAAAGGAGAAGAACT) and P2GFPEcoRI (CCGGAATTCTTACCACTCCAGATTTTGCTGTTCTGTT GATTCTGGCGAGGCATTTTTCGCTTTGTATAGTTCATCCATGC) or P2GHEcoRI (CC GGAATTCTTATTTGTATAGTTCATCCATGC) were used to amplify GFP from pUA66. The PCR products were digested with *Xba*I and *Eco*RI and ligated into *Xba*I and *Eco*RI sites of pBRlac_i_tac creating pBR*hipB* or pBR*hipB72* and pBRGFP or pBRGFP-H, respectively.

### Mutant generation

The HipB(W88A) mutant and the truncation mutant HipB72 (deletion of all amino acid residues after position 72) were generated using site directed mutagenesis. Primers used for the mutagenesis are as follows:

5′- AACCTGGAAGCGTAACTCGAGGATCCG and

5′- CGGATCCTCGAGTTACGCTTCCAGGTT for HipB(W88),

5′-GGACCGCATATGAGTTTTCAGAAAATCTATAGTCC and

5′-GAACCGCTCGAGTTAATCACACAGCGTCATCGAC for HipB72). The generation of mutants was verified by DNA sequencing.

### Strain construction

Precise deletion-replacement of *hslVU* was created by the method of Datsenko and Wanner [50].

### Protein expression and purification

N-terminally hexa-histidine (his_6_)-tagged HipB, HipB72, HipA and Lon were purified using strains and plasmids indicated in Table 2. Strains were induced by addition of 0.2% arabinose or 1 mM IPTG for 4 h. Cells were lysed by sonication in the presence of 1 mg•ml^−1^ lysozyme. Protein extracts were applied to a Ni-NTA resin (QIAGEN). The columns were washed with buffer containing 20 mM imidazole, and eluted with buffer containing 250 mM imidazole. The eluted protein was concentrated and dialyzed with protein storage buffer (50 mM Tris-HCl, pH 8, 250 mM NaCl, 5 mM dithiothreitol [DTT], 5% glycerol) [51].

Proteins used for fluorescence polarization-based binding assays were expressed and purified as described below. Plasmid DNA (pET28a-HipA or pET15b-HipB) was transformed into BL21(DE3)pLysS competent cells, which were plated onto LB agar containing 50 µg•mL^−1^ kanamycin for HipA expressing cells and 100 µg•mL^1^ ampicillin for HipB expressing cells, respectively. All cells were allowed to grow at 37°C with appropriate antibiotics. A single colony was selected from each plate and inoculated into 250 mL LB for overnight growth. 40 mL cells were transferred into 1.5 L LB and were incubated until the OD_600_ reached 0.6. Proteins were induced for four hours by adding 1 mM IPTG. Cells were harvested by centrifugation at 4,000× g, 4°C for 10 minutes and the harvested cells were stored in −80°C. The frozen cells were thawed and resuspended in 50 mL buffer A (20 mM Tris-HCl pH 7.5, 300 mM NaCl, 5% glycerol) containing one protease inhibitor tablet and 20 µg•mL^−1^ DNase I (Roche). After homogenizing the cells with a dounce tissue grinder, cells were disrupted using a microfluidizer (model M110L, Microfluidics) and centrifuged at 31,000× g, 4°C for 1 hour. The supernatant was applied to 3 mL Ni-NTA affinity resin equilibrated with Buffer A and allowed to run by gravity. The column was washed overnight with buffer A containing 20 mM imidazole at 0.5 mL•min^−1^ flow rate. Proteins were eluted with 50–200 mM imidazole gradient. Protein homogeneity was estimated by a SDS-PAGE gel with coomassie staining. Buffer exchange and protein concentration were achieved using Amicon ultra centrifugal filter (Millipore). Protein concentrations were determined by Bradford using BSA as the standard.

### Fluorescence polarization-based binding affinity assays

Fluorescence polarization experiments were performed to determine the binding affinities of HipB, the HipB(W88A) mutant and HipB72 proteins for DNA and HipA [52]. A DNA oligodeoxynucleotide containing two *hipB* operator sites (*O_1_O_2_*) with the sequence

(5′-TTATCCGCTTAAGGGGATATTATAAGTTTTATCCTTTAGTGAGGATAA-3′) and labeled with 6-carboxyfluorescein at 5′-end was purchased (Integrated DNA Technologies Inc) and used without further purification. The labeled DNA was heated to 95°C for two to five minutes in the presence of an equal amount of the unlabelled complementary strand. The strands were annealed by cooling slowly at room temperature. DNA binding was carried out in 0.5 mL binding buffer A (20 mM Tris-HCl pH 7.5, 100 mM NaCl and 5% glycerol) in which 1.0 nM labeled DNA was included. Changes in fluorescence polarization were monitored using a Panvera Beacon 2000 (Panvera Corporation) as a function of increasing concentration of HipB. Binding was done at 10°C. The binding affinity of HipA for HipB was determined by titrating HipA into a solution of 50 nM HipB, which was pre-incubated with 1 nM labeled DNA for 2 minutes, in 0.5 mL binding buffer A. The concentration of HipB assured the stoichiometric binding of the *O_1_O_2_* DNA site, as 50 nM HipB is nearly 100 fold more than the K_d_ that was determined for HipB-*O_1_O_2_* binding. The wavelengths used for excitation and emission were 490 nm and 530 nm, respectively. Dissociation constants were obtained as described previously [52]. Briefly, the increase in fluorescence millipolarization (mP=P·10^−3^, where P is polarization) as a function of the increasing HipB or HipA concentration is fit using the curve fitting function of Kaleidagraph and the equation: P={(P_bound_−P_free_) [HipB-DNA]/(K_d_+[HipB-DNA])}+P_free_, where P is the polarization measured at a given concentration of HipB, P_free_ is the initial polarization of the free DNA, and P_bound_ is the maximum polarization of specifically bound DNA. For determination of the HipA affinity for HipB, the same equation is used but the HipB-DNA term is replaced by HipA-(HipB-DNA). Nonlinear least squares analysis is used to determine P_bound_ and K_d_.

### *In vivo* degradation and Western Blot analysis

The rate of degradation of HipB, HipB72, GFP and GFP-H was determined using samples from exponentially growing cells. Expression of proteins from pBR*lac_i_tac* was induced by addition of 1 mM IPTG at OD_600_ of 0.3. After 1 h induction, protein synthesis was inhibited by the addition of 100 µg/ml Cam, and samples were removed at indicated time points. Same sample volume was loaded in each well of an SDS-PAGE gel. Protein levels were detected by Western blotting using either a monoclonal His-tag antibody (EMD Biosciences) or polyclonal antibody to GFP (Abcam) and a polyclonal goat-anti mouse IgG AP conjugate.

### *In vitro* degradation assay

To monitor degradation of HipB, His_6_-Lon (0.6 µM) and His_6_-HipB (0.48 µM) or His_6_-HipB72 (0.48 µM) were added to a degradation buffer [50 mM Tris-HCl (pH 8.0), 4 mM ATP, 7.5 mM MgCl_2_] and incubated at 37 °C. When indicated, His_6_-HipB was mixed with either His_6_-HipA (0.48 µM) lysozyme (0.48 µM) or duplex DNA containing a 21 bp *hipB* operator site (0.24 µM) (sequence of the top strand ACTATCCCCTTAAGGGGATAG) or a control deoxynucleotide (sequence of the top strand ATGATGAGCTTTCAGAAGATC). Samples were removed at indicated times, and analyzed by SDS-PAGE. The same sample volume was loaded in each well.
