# Correction: Regulation of the *Escherichia coli* HipBA Toxin-Antitoxin System by Proteolysis

**DOI:** 10.1371/annotation/e608601c-eadd-4c11-adb2-7b605aba9c44

**Published:** 2012-09-17

**Authors:** Sonja Hansen, Marin Vulić, Jungki Min, Tien-Jui Yen, Maria A. Schumacher, Richard G. Brennan, Kim Lewis

There was an error in the placement of Figures 6 and 7. The figures were switched. The legends are in the correct locations. 

